# Iridoids in gardenia fruit: key components in liver disease therapy

**DOI:** 10.3389/fnut.2025.1667863

**Published:** 2025-09-25

**Authors:** Qian Cao, Xinyi Du, Anli Liu, Qinggui Li, Qingqing Luo, Yunsong Chen, Rong Wang, Lingjie Meng

**Affiliations:** ^1^Institute of Life Sciences, Zunyi Medical University, Zunyi, Guizhou, China; ^2^College of Basic Medicine, Zunyi Medical University, Zunyi, Guizhou, China; ^3^The Key Lab of Guizhou Provincial Department of Education for Medical Prevention and Treatment of Tumor, Zunyi Medical University, Zunyi, Guizhou, China

**Keywords:** gardenia fruit, iridoids, liver disease, pharmacological effects, molecular mechanism

## Abstract

Liver diseases pose a serious threat to human health, necessitating the development of safe and effective preventive and therapeutic strategies. Gardenia fruit (GF), the mature fruit of *Gardenia jasminoides* Ellis, has been widely used in both food and medicinal applications. Over time, GF and its major bioactive constituents, the iridoids, have demonstrated significant potential in the prevention and treatment of various liver diseases. This review first summarizes the structural characteristics and pharmacological activities of the major iridoids in GF from a phytochemical perspective. It then focuses on the therapeutic effects of GF extracts against non-alcoholic fatty liver disease, cholestatic liver disease, acute liver injury, and liver fibrosis. Furthermore, the review provides a comprehensive examination of the multi-target mechanisms by which iridoids mediate their hepatoprotective effects. These mechanisms include the regulation of lipid metabolism, attenuation of cholestasis, suppression of inflammation and oxidative stress, amelioration of mitochondrial dysfunction, modulation of autophagy, as well as anti-fibrotic, anti-hepatocarcinogenic, and detoxification activities. Among these, the inhibition of inflammation and oxidative stress is highlighted as a primary mechanism of action. In addition, this review critically evaluates the current limitations associated with the use of GF and its iridoids in liver disease treatment and discusses potential directions for future research. The aim of this review is to provide theoretical foundations and scientific guidance for the further research and development of GF-based therapeutic agents.

## 1 Introduction

The liver is an essential organ in the human body, playing a critical role in detoxification, metabolism regulation, protein synthesis, bile production, and energy storage (1). Various factors, including viruses, bacteria, chemical substances, and medications, can cause different degrees of liver injury (2). Prolonged exposure to these toxic elements can precipitate the development and progression of various liver diseases, such as viral hepatitis, alcoholic liver diseases (ALD), non-alcoholic fatty liver diseases (NAFLD), and autoimmune liver diseases (AILD) (3). If these initial pathological changes are not addressed promptly and effectively, they may progressively worsen over time, ultimately leading to irreversible severe liver conditions like cirrhosis and hepatocellular carcinoma (HCC). In the past few decades, liver diseases and their related complications have become a major global health issue, causing more than two million deaths each year, which represents about 4% of worldwide mortality (4). Therefore, identifying safe and effective treatments to prevent or reverse liver diseases is essential for addressing this significant public health concern.

Gardenia fruit (GF), the mature fruit of *Gardenia jasminoides* Ellis, is referred to as “Zhizi” in Chinese, “Cape Jasmine” in Korean, and “Sanshishi” in Japanese, and was initially documented in the *Shennong Herbal Classic* (5). In traditional Chinese medicine (TCM), GF has been utilized for its properties in clearing heat and purging fire, promoting diuresis to clear heat, and cooling the blood while detoxifying (6). As a clinically important hepatoprotective and cholagogic agent, GF is frequently utilized in various classic formulations, such as Zhi Zi Da Huang Tang (7), Yin Chen Hao Tang (8), Qushi Huayu Decoction (9) and Zhizi Baipi Soup (10) for the treatment of liver diseases. Studies in pharmacology have shown that GF has multiple effects, such as reducing inflammation (11), antidepressant (12), antiviral (13), anti-thrombotic effect (14), and hepatoprotective activities (15). Notably, GF has been officially acknowledged by China's Ministry of Health as part of the initial group of medicinal and edible resource varieties. In China and East Asia, GF is widely used both as a functional food supplement, which are incorporated as food ingredients and dietary supplements (16). Moreover, in both United States and the European Union, GF is commercially turned into concentrated fruit juices, either on its own or mixed with other fruits, for various uses (17). Phytochemical studies have demonstrated that iridoids (IGs) are characteristic constituents of GF and are generally considered the functional components responsible for its pharmacological activities (18). Importantly, IGs exhibit considerable potential in the prevention and treatment of liver diseases.

To date, a considerable body of research has explored the role of iridoids derived from GF in the prevention and treatment of liver diseases. These studies underscore the significant potential of these compounds and provide a scientific foundation for identifying the constituents responsible for their hepatoprotective effects. Nonetheless, the existing literature is fragmented and lacks a systematic review and synthesis. Previous reviews have predominantly concentrated on the phytochemical composition and general pharmacological properties of GF, with insufficient focus on the hepatoprotective mechanisms of its iridoids. Consequently, these reviews are not comprehensive and do not adequately reflect recent advancements in the field. To address this deficiency, we conducted an exhaustive search of databases, including PubMed, ScienceDirect, Elsevier and Google Scholar and CNKI, to collect all pertinent literature published up to 2024 concerning the use of GF and its active iridoid components in liver diseases prevention and treatment. This review is the first to systematically summarize the therapeutic effects and underlying mechanisms of iridoids in liver diseases, thereby establishing a solid scientific foundation for the future development of GF- or iridoid-based hepatoprotective drugs or functional foods.

## 2 IGs in GF

IGs are active compounds commonly found in the Scrophulariaceae, Pyrolaceae, Oleaceae, Labiatae, Rubiaceae, and Gentianaceae families (19). These compounds typically occur as glycosides with a glucose unit attached at C-1. IGs are structurally divided into two main types: carbocyclic iridoids, which have a cyclopentane ring connected to a dihydropyran unit, and secoiridoids, formed by the breaking of the cyclopentane ring. IGs generally have significant bitter properties. For instance, iridoid compounds, the primary element of gentian root, serve as a crucial ingredient in the creation of bitter medications and also enhance the secretion of gastric juice and bile. Therefore, they are used in traditional medicine to treat liver diseases (20).

**Figure 1:**
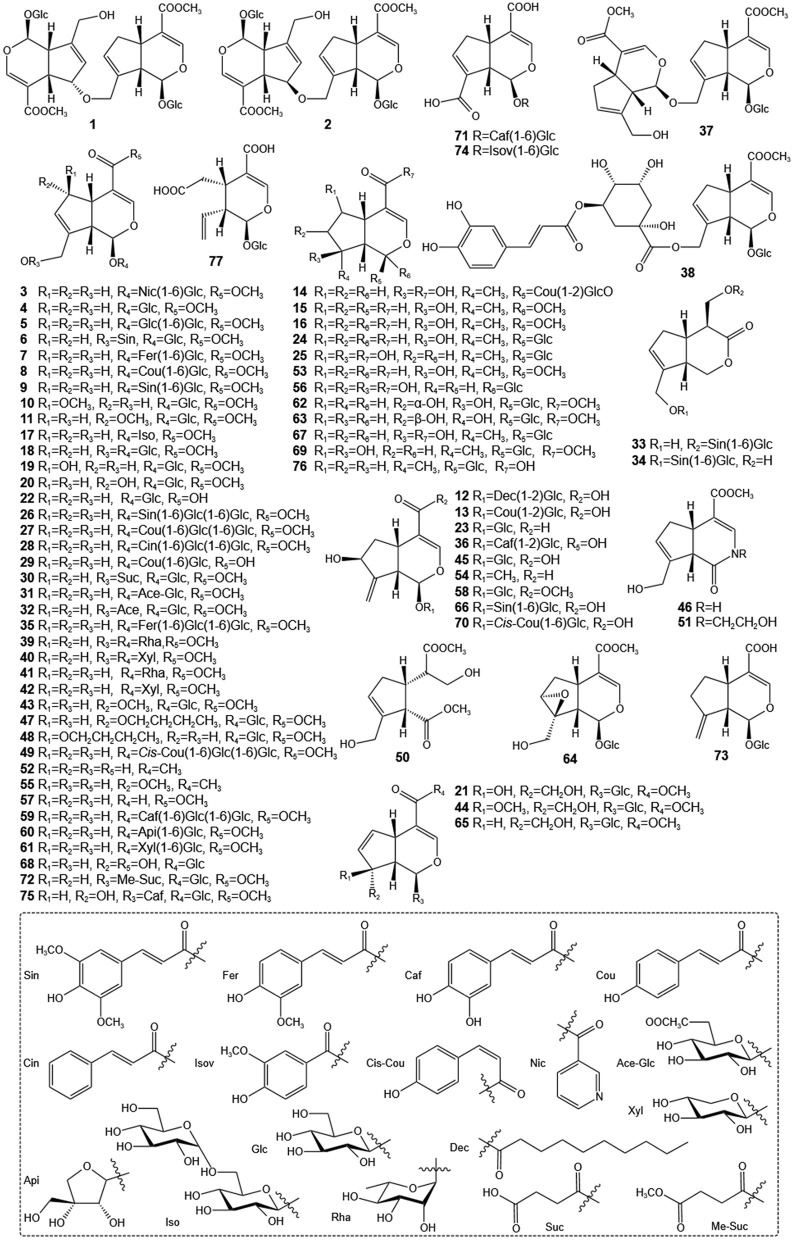
Structures of iridoids of GF.

Since the initial discovery of geniposide and gardenoside in the 1960s, researchers have identified many other IGs from GF, such as geniposidic acid, genipin, and genipin-1-β-gentiobioside (21, 22). Among these, geniposide stands out as a key IG, with its content ranging from about 3.18%−6.32% in the whole fruit and reaching up to 7.68% in the seeds (23, 24). The geniposide content in GF exhibits considerable variability across different geographical regions. Shang et al. (25) reported that the highest geniposide concentration was found in samples from Hunan province (34.64 ± 0.45 mg/g), followed by those from Jiangxi (33.10 ± 0.36 mg/g), Anhui (30.73 ± 0.41 mg/g), Sichuan (27.96 ± 0.45 mg/g), and Henan (27.88 ± 0.37 mg/g) provinces. Furthermore, Xu et al. (26) documented significant variations in the concentrations of 12 representative components across 40 samples, with geniposide levels ranging from 37.92 to 72.23 mg/g and the total content of seven iridoids varying between 59.93 and 94.31 mg/g. A more recent investigation quantified 13 major chemical constituents in GF. This study revealed that the concentrations of six iridoids—geniposide, genipin, shanzhiside, geniposidic acid, genipin 1-gentiobioside, and deacetylasperulosidic acid methyl ester—achieved maximum values of 104.63 ± 17.68, 0.27 ± 0.01, 3.22 ± 0.09, 0.412 ± 0.02, 19.08 ± 0.48, and 2.98 ± 0.70 mg/g, respectively (27). Notably, geniposide can be enzymatically converted into genipin—a compound utilized as a natural red/blue colorant in the food industry upon reaction with different amino acids—through the action of β-D-glucosidase from gut microbiota. Pharmacological studies have demonstrated that IGs exhibit notable multifaceted biological activities, including hepatoprotective (28), anti-inflammatory (29), neuroprotective (30), antitumor (31), hypoglycemic and hypolipidemic activities (32). The diverse pharmacological effects of IGs are primarily attributed to modifications such as epoxidation and hydroxylation of their fundamental structure, as well as esterification of aromatic acids derived from the shikimic acid pathway (33). The IGs components in GF are compiled and summarized in Table 1, with their chemical structures illustrated in Figure 1.

**Table 1:** Chemical information of iridoids from gardenia fruit.

**No**	**Compound name**	**Formula**	**Molecular weight**	**References**
1	disperoside A	C_34_H_46_O_20_	774.26	(34)
2	disperoside B	C_34_H_46_O_20_	774.26	(34)
3	6′-nicotinoyloxygeniposide	C_23_H_27_NO_11_	493.16	(34)
4	geniposide	C_17_H_24_O_10_	388.14	(34)
5	genipin-gentiobioside	C_23_H_34_O_15_	550.19	(34)
6	10-*O*-*trans*-sinapoylgeniposide	C_28_H_34_O_14_	594.19	(34)
7	lippianoside B	C_27_H_32_O_13_	564.54	(34)
8	6′-*O*-*trans*-*p*-coumaroylgeniposide	C_26_H_30_O_12_	534.17	(34)
9	6′-*O*-*trans*-sinapoylgeniposide	C_28_H_34_O_14_	594.19	(34)
10	6-*O*-methyldeacetylasperulosidic acid methyl ester	C_18_H_26_O_11_	418.15	(34)
11	6-*O*-methylscandoside methyl ester	C_18_H_26_O_11_	418.15	(34)
12	2′-*O*-decanoylgardoside	C_26_H_40_O_11_	528.26	(34)
13	2′-*O*-*trans*-*p*-coumaroylgardoside	C_25_H_28_O_12_	520.16	(34)
14	2′-*O*-(4-methoxycinnamoyl)mussaenosidic acid	C_25_H_30_O_12_	522.17	(34)
15	euphrasin	C_21_H_16_O_4_	212.10	(34)
16	campsinol	C_21_H_16_O_4_	212.10	(34)
17	genipin 1-*O-β*-D-isomaltoside	C_23_H_34_O_15_	550.19	(35)
18	genipin 1,10-di-*O-β*-D-glucopyranoside	C_23_H_34_O_15_	550.19	(35)
19	scandoside methyl ester	C_17_H_24_O_11_	404.13	(35)
20	deacetylasperulosidic acid methyl ester	C_17_H_24_O_11_	404.13	(35)
21	gardenoside	C_17_H_24_O_11_	404.13	(35)
22	geniposidic acid	C_16_H_22_O_10_	374.12	(36)
23	gardaloside	C_16_H_22_O_9_	358.13	(37)
24	ixoroside	C_16_H_24_O_9_	360.14	(37)
25	shanzhiside	C_16_H_24_O_11_	392.13	(37)
26	6″-*O-trans*-sinapoylgenipin gentiobioside	C_34_H_44_O_19_	756.25	(38)
27	6″-*O-trans*-*p*-coumaroylgenipin gentiobioside	C_32_H_40_O_17_	696.23	(38)
28	6″-*O-trans*-cinnamoylgenipin gentiobioside	C_32_H_40_O_16_	680.23	(38)
29	6′-*O-trans*-*p*-coumaroylgeniposidic acid	C_25_H_28_O_12_	520.16	(38)
30	10-*O*-succinoylgeniposide	C_21_H_28_O_13_	488.15	(38)
31	6′-*O*-acetylgeniposide	C_19_H_26_O_11_	430.15	(38)
32	10-*O*-acetylgeniposide	C_19_H_26_O_11_	430.15	(38)
33	11-(6-*O-trans*-sinapoylglucopyranosyl)gardendiol	C_27_H_34_O_13_	566.20	(38)
34	10-(6-*O-trans*-sinapoylglucopyranosyl)gardendiol	C_27_H_34_O_13_	566.20	(38)
35	6″-*O-trans*-feruloylgenipin gentiobioside	C_33_H_42_O_18_	726.24	(39)
36	2′-*O-trans-*caffeoylgardoside	C_25_H_28_O_13_	536.15	(39)
37	jasmigeniposide B	C_28_H_36_O_14_	596.21	(39)
38	jasmigeniposide A	C_33_H_40_O_18_	724.22	(39)
39	genipin 1,10-di-*O*-α-L-rhamnoside	C_23_H_34_O_13_	518.20	(40)
40	genipin 1,10-di-*O*-β-D-xylopyranoside	C_21_H_30_O_13_	490.17	(40)
41	genipin 1-*O*-α-L-rhamnoside	C_17_H_24_O_9_	372.14	(40)
42	genipin 1-*O*-β-D-xylopyranoside	C_16_H_22_O_9_	358.13	(40)
43	6-*O*-methylscandoside methyl ester	C_18_H_26_O_11_	418.15	(41)
44	8-*O*-methylmonotropein methyl ester	C_18_H_26_O_11_	418.15	(41)
45	gardoside	C_16_H_22_O_10_	374.12	(41)
46	gardenamide	C_11_H_13_NO_4_	223.08	(42)
47	6α-butoxygeniposid	C_21_H_32_O_11_	460.19	(42)
48	6β-butoxygeniposid	C_21_H_32_O_11_	460.19	(42)
49	6″-*O*-*p*-*cis*-coumaroylgenipin gentiobioside	C_32_H_40_O_17_	696.23	(42)
50	gardenate A	C_12_H_18_O_6_	258.11	(43)
51	2-hydroxyethyl gardenamide A	C_13_H_17_NO_5_	267.11	(43)
52	gardenal-I	C_11_H_14_O_4_	210.09	(44)
53	gardenal-II	C_11_H_16_O_4_	212.10	(44)
54	gardenal-III	C_11_H_14_O_4_	212.10	(44)
55	6-α-methoxy geniposide	C_18_H_26_O_11_	418.15	(44)
56	lamalbidic acid	C_15_H_22_O_12_	394.11	(44)
57	genipin	C_11_H_14_O_5_	226.08	(45)
58	gardoside methyl ester	C_17_H_24_O_10_	388.14	(46)
59	6″-*O-*trans-caffeoylgenipin gentiobioside	C_32_H_40_O_18_	712.22	(47)
60	genipin 1-O-β-D-apiofuranosyl (1 → 6) *β-*D-glucopyranoside	C_22_H_32_O_14_	520.18	(47)
61	genipin 1-O-α-D-xylopyranosyl (1 → 6) β-D-glucoopyranoside	C_22_H_32_O_14_	520.18	(47)
62	genameside A	C_17_H_26_O_12_	422.14	(48)
63	genameside B	C_17_H_26_O_12_	422.14	(48)
64	7α,8β-epoxy-8α-dihydrogeniposide	C_17_H_24_O_11_	404.13	(49)
65	8-epiapodantheroside	C_17_H_24_O_10_	388.14	(49)
66	6′-*O-trans*-sinapoyl gardoside	C_27_H_32_O_14_	580.18	(49)
67	mussaenosidic acid	C_16_H_24_O_10_	376.14	(50)
68	deacetylasperulosidic acid	C_16_H_22_O_11_	390.12	(50)
69	shanzhisidemethyl ester	C_17_H_26_O_11_	406.15	(45)
70	2′-*O*-*cis*-coumaroylgardoside	C_25_H_28_O_12_	520.16	(51)
71	6′-*O*-caffeoylioxide	C_25_H_26_O_14_	550.13	(51)
72	10-*O*-(4″-*O*-methylsuccinoyl)geniposide	C_22_H_30_O_13_	502.17	(52)
73	7-deoxygardoside	C_16_H_22_O_9_	358.13	(53)
74	tarenninoside C	C_24_H_26_O_14_	538.13	(53)
75	10-*O*-caffeoyl deacetyl daphylloside	C_26_H_30_O_14_	566.16	(53)
76	7-deoxy-8-epiloganic acid	C_16_H_24_O_9_	360.14	(53)
77	secologanoside	C_16_H_22_O_11_	390.12	(53)

## 3 GF is utilized in treating various liver diseases

### 3.1 Nonalcoholic fatty liver disease (NAFLD)

NAFLD is a prevalent disorder characterized by an abnormal buildup of fat in the liver, progressing from simple fatty liver to non-alcoholic steatohepatitis (NASH), potentially resulting in fibrosis, cirrhosis, and liver cancer (54). It is closely associated with metabolic syndrome, obesity, type 2 diabetes, and dyslipidemia, making it a major global health issue (55). The complex causes of NAFLD include genetic factors, insulin resistance, adipose tissue dysfunction, and oxidative stress (56). Recent research suggested that gut microbiota and its metabolites play a role in NAFLD's development and progression, with dysbiosis potentially causing liver inflammation and damage (57). Moreover, endothelial cell dysfunction is also linked to NAFLD progression and may increase cardiovascular disease risk (58). Although there is no FDA-approved medication for NAFLD, managing the condition primarily relies on diet and weight loss. New treatments are being developed to target specific metabolic pathways to change the course of the disease.

GF, a traditional Chinese medicine clinically utilized for NAFLD treatment, significantly improved metabolic and hepatic parameters in high-fat diet (HFD)-induced models. Current research has shown that relative to the non-treated HFD controls, GF (25, 50, 100 mg/kg) administration significantly lowered concentrations of serum total cholesterol (TC), lipoprotein cholesterol, triglycerides (TG), ALT, AST, LDH, free fatty acids (FFA), glucose, and insulin, concurrently reducing hepatic TG, TC, and malondialdehyde (MDA) levels (59). Furthermore, the aqueous extract of GF (28 mg/kg) modulate key liver injury biomarkers and signaling pathways—including mTOR, 8-hydroxy2′-deoxyguanosine (8-OHdG), TGF-β, ERK1/2 phosphorylation, and oxidative stress markers (60). In addition, the crude extract of crocin from GF (100 and 200 mg/kg) also attenuated hyperglycemia, dyslipidemia, and hepatic oxidative stress, while beneficially restructuring gut microbiota in HFD-fed rats by reducing the Firmicutes/Bacteroidetes ratio and enriching *Akkermansia, Bacteroides*, and *Lactobacillus* (61). Notably, GF-containing TCM formulas effectively treat NAFLD in clinical and preclinical settings. Yin Zhi Huang (YZH, 10 and 30 ml/kg daily) reduces diet-induced obesity and liver fat by inhibiting AMPK/SREBP-1-related lipogenesis and boosting AMPK/ACC/CPT1A-driven mitochondrial β-oxidation (62). Likewise, Qushi Huayu Decoction (QHD) reduces liver lipogenesis through XBP1s-dependent pathways, circumventing the regulatory influence of SREBP1 and ChREBP (63).

### 3.2 Cholestatic liver disease (CLD)

CLD involves conditions affecting the bile ducts, caused by primary or secondary injuries (64). Its multifactorial etiology includes immune, genetic, and environmental factors. CLD progression varies, often involving ductular reaction, hepatic fibrosis, bile acid accumulation, inflammatory infiltration, and potential intestinal barrier impairment (65). Bile acids are crucial for cholesterol elimination, and disruptions in their synthesis and transport can lead to CLD by causing toxic substance retention (66). It has been reported that the aqueous extract of GF (21 and 42 mg/kg) alleviated alpha-naphthylisothiocyanate (ANIT)-induced hepatotoxicity and cholestasis in rats. This protective effect was associated with the upregulation of *Cyp8b1* expression, which inhibited BA synthesis in the liver, promoted BA excretion via the intestinal-fecal route, and enhanced the enterohepatic circulation of Bas (67). At the same time, the GF aqueous extract (900 mg/kg) restored disturbances in primary BA biosynthesis, glycerophospholipid metabolism, tryptophan metabolism, and arachidonic acid metabolism caused by ANIT administration (68). In addition, Yinchenhao Decoction (YCHD), a famous traditional Chinese formula containing GF (6, 9 and 12 g/kg), has been shown to modulate the expression of metabolic enzymes and transporters under cholestatic conditions (69). Notably, herbal formulations follow specific compatibility principles, as interactions between herbs can lead to either synergistic or antagonistic effects. Indeed, studies have demonstrated that the combination of rhubarb and gardenia exerts synergistic effects in ANIT-induced cholestatic rats at both pharmacodynamic and pharmacokinetic levels (70).

### 3.3 Acute liver injury (ALI)

ALI is a clinical syndrome characterized by significant hepatic damage, involving extensive infiltration of inflammatory cells, structural destruction, and functional abnormalities of the liver. This condition can be precipitated by various etiological factors, including excessive alcohol consumption, viral infections, medication overdoses, and acute exposure to toxins, potentially progressing to liver failure in severe cases (71). Previous research has indicated that treatment with 50% ethanol extract of GF (880 mg/kg) may mitigate acetaminophen (APAP)-induced hepatotoxicity, likely due to its anti-inflammatory and antioxidant properties (72). Additionally, Liu et al. (73) reported that YCHT (8 g/kg) provides liver protection by influencing metabolic pathways and changes in gut microbiota during liver damage caused by CCl_4_. Moreover, GF aqueous extracts (112.5, 225 and 450 mg/ml) have demonstrated efficacy in reducing Bacillus Calmette-Guérin (BCG)- and lipopolysaccharide (LPS)-induced immunological liver injury in murine models (74). Furthermore, Zhizi Baipi Soup (ZBS) and its simplified formulations containing Zhizi exhibit significant protective effects against concanavalin A (Con A)-induced immunological liver injury in mice (75).

### 3.4 Liver fibrosis (LF)

LF is a significant pathological state marked by the overaccumulation of extracellular matrix (ECM) proteins, leading to the disruption of normal liver architecture and function. LF, which can arise from various types of liver damage, may advance to cirrhosis, liver failure, and HCC, creating a major global health challenge (76). Central to this fibrogenic process is the activation of hepatic stellate cells (HSCs), which undergo transdifferentiation into myofibroblasts and become the primary source of excessive ECM deposition (77). Previously, Shin et al. (78). reported that the aqueous extracts of GF (200 mg/kg) mitigate thioacetamide-induced LF in mice through the AMPK/SIRT1/NF-κB and Nrf2 signaling pathway. Similarly, GF root aqueous extracts (5, 10 and 20 mg/kg) also conferred protection against CCl_4_-induced LF in rats, potentially through mechanisms involving enhanced ECM degradation and inhibition of lipid peroxidation (79). In addition, YCHD (3.15 g/kg) alleviated dimethylnitrosamine (DMN)-induced LF by modulating enzymes involved in bile acid metabolism and inhibiting chenodeoxycholic acid (CDCA)-induced HSC proliferation and activation via the TGF-β1/Smad/ERK signaling pathway (80). Furthermore, synergistic approaches show promise: Co-treatment with GF and Silymarin effectively ameliorated oxidative stress-driven LF in a thioacetamide mice model, attributed to the regulation of hepatic sirtuin1 activity (81). Additionally, Zhizi Bopi Decoction and its individual components have also been reported to exhibit significant, albeit varying degrees of, anti-fibrotic effects on the liver (82).

## 4 The role and mechanism of iridoids from GF in the treatment of liver diseases

### 4.1 Effects of iridoids on hepatic lipid metabolism

#### 4.1.1 Nuclear factor erythroid 2-related factor 2 (Nrf2) signaling pathway

The Nrf2 signaling pathway is crucial for defending cells against oxidative stress and inflammation. As a transcription factor, Nrf2 moves to the nucleus upon activation and binds with the antioxidant response element in gene promoters, triggering the production of antioxidant and protective enzymes (83, 84). Normally, Kelch-like ECH-associated protein 1 (Keap1) regulates this pathway by holding Nrf2 in the cytoplasm and promoting its degradation. However, during oxidative stress, Nrf2 detaches from Keap1, allowing the activation of genes that detoxify and eliminate harmful oxidants and electrophiles (85). As early as 2019, Shen et al. (86) found that geniposide (50, 75 and 100 mg/kg) exerts protective effects against lipid accumulation by enhancing antioxidant and anti-inflammatory capacities, which is at least partly attributed to its inhibition of the Nrf2/HO-1 pathway.

#### 4.1.2 Adenosine monophosphate-activated protein kinase (AMPK) signaling pathway

AMPK serves as a pivotal cellular energy sensor essential for maintaining energy homeostasis. Upon activation by an elevated AMP/ATP ratio during energy stress, AMPK coordinates metabolic changes by enhancing catabolic pathways that generate ATP and inhibiting anabolic processes that consume ATP (87, 88). Importantly, AMPK regulates lipid, glucose, and protein metabolism, autophagy, and mitochondrial function, underscoring its critical role in addressing metabolic disorders such as NAFLD (89). It is noteworthy that in addition to the Nrf2/HO-1 pathway, geniposide also suppresses the AMPK pathway to inhibit lipid accumulation (86). Additionally, a combination of geniposide, Peanut Skin Extract (PSE), and Isoquercitrin has been shown to significantly reduce body and liver weight, ameliorate hepatic steatosis, and improve liver function markers in mice. These effects are primarily mediated through regulation of the AMPK/ACC/CPT1 and AMPK/ULK1/LC3B signaling pathways (90).

#### 4.1.3 MicroRNAs (miRNAs)

miRNAs are small noncoding RNAs, usually around 22 nucleotides long, that are essential for regulating gene expression after transcription. These molecules are highly conserved across species and are involved in a broad range of biological activities, such as cell growth, differentiation, development, and programmed cell death (91). The abnormal regulation of miRNAs has been linked to the development of several diseases, including cancer, cardiovascular diseases, and neurodegenerative disorders (92). It has been reported that genipin (5 and 20 mg/kg) reduces HFD-induced hyperlipidemia and liver steatosis in mice by modulating the miR-142a-5p/SREBP-1c pathway (93). At the same time, the researchers further found that genipin (20 mg/kg) demonstrated wide-ranging benefits in ameliorating metabolic disorders and sperm dysfunction caused by a high-fat diet in male mice by regulating miR-132 in a tissue-specific manner (94). These findings highlight the significant roles of iridoids and miRNAs in preventing and treating hepatic lipid metabolism issues.

#### 4.1.4 Intestinal microbiota

The intestinal microbiota is integral to the maintenance of gastrointestinal homeostasis and overall health. This intricate ecosystem, comprising bacteria, viruses, fungi, and protozoa, inhabits the human gut and participates in different bodily functions such as digestion, metabolism, and immune response. The composition and diversity of the intestinal microbiota are modulated by factors such as diet, genetics, and environmental exposures, with perturbations in this microbial community having significant implications (95). The gut-liver axis represents a pivotal pathway through which the gut microbiota exerts influence on hepatic lipid metabolism, and disruptions within this axis are associated with metabolic disorders such as NAFLD and obesity (96, 97). Notably, geniposide-based combination therapies demonstrate efficacy in regulating hepatic lipid metabolism through intestinal microbiota modulation. This is evidenced by Peng et al. (98) who reported that a geniposide and chlorogenic acid combination ameliorates NASH in HFD mice, partly via protection of gut barrier function. Recent study further confirmed that this combination reduces blood lipids and hepatic lipid accumulation, lowers serum ALT/AST levels and liver weight index, ameliorates intestinal dysbiosis, and modulates intestinal and serum bile acid metabolism in murine NASH models (99).

#### 4.1.5 Pyroptosis

Pyroptosis, an inflammatory form of programmed cell death mediated by gasdermin proteins, functions as a vital defense mechanism against microbial infections by activating inflammasomes—multiprotein complexes responsible for detecting pathogens and cellular stress. This process initiates the activation of inflammatory caspases, leading to the elimination of infected cells and the recruitment of immune effectors to enhance immune responses (100, 101). Nevertheless, dysregulated pyroptosis can contribute to inflammatory pathology and tissue damage, highlighting its dual roles in health and disease (102). Relevant results showed that, in both *in vitro* (10, 25 and 50 μM) and *in vivo* (40 mg/kg) models of NAFLD, gardenoside attenuates lipid accumulation, enhances cell viability, reduces ROS, and suppresses pyroptosis via downregulation of pyroptosis markers (NLRP3, ASC, caspase-1 p20, GSDMD-N, IL-1β) and reduced CTCF/DPP4 expression (103). While genipin's role in hepatic lipid metabolism regulation is established, its relationship with pyroptosis remains undefined. Study demonstrated that genipin attenuates HFD-induced liver damage and inhibits UCP2-mediated pyroptosis in both HFD-fed mice (5 and 20 mg/kg) and free fatty acid (FFA)-treated hepatocytes (20 μM) models exhibiting marked pyroptotic activation (104).

#### 4.1.6 Apoptosis

Apoptosis is a crucial programmed cell death process that maintains homeostasis in multicellular organisms by removing unfit or damaged cells without causing inflammation. It is vital from development through adulthood, involving complex molecular pathways governed by Bcl-2 proteins, caspases, and their inhibitors (105). Recent studies suggested that geniposide (900 μg/ml) influences global DNA methylation levels and modulates the expression of P53, Bcl-2, and Akt, thereby collectively inhibiting apoptosis in human hepatocytes (106).

### 4.2 Effects of iridoids on cholestasis

#### 4.2.1 Bile acids (BAs) homeostasis

BAs are crucial for lipid digestion, absorption, and act as signaling molecules in metabolic regulation. Their homeostasis involves liver synthesis, gut microbiota modification, and intestinal reabsorption, essential for metabolic and immune balance (107). Disruptions can cause diseases like liver disorders and metabolic syndrome. BA regulation involves pathways and receptors like farnesoid X receptor (FXR) and Takeda G-protein-coupled receptor 5 (TGR5), which influence glucose, lipid, and energy metabolism (108). FXR is particularly important for BA synthesis and transport, with its deficiency potentially leading to metabolic disorders (109). Notably, among the iridoids of GF, geniposide is the primary compound that affects cholestasis by modulating BA metabolism. In one recent study, geniposide (50 mg/kg) regulated cholesterol metabolism by modulating the liver-gut crosstalk of bile acids mediated by FXR in C57BL/6 and ApoE-/- mice. This effect promoted hepatic BA synthesis and ileal BA excretion by regulating the enterohepatic circulation of BAs (110). In addition, in cases of cholestatic liver injury, geniposide (50 and 100 mg/kg) consistently regulates BA transporters in an FXR-dependent manner. It alleviates sclerosing cholangitis by upregulating canalicular efflux transporters (BSEP, MRP2, MDR1, MDR2) to enhance biliary BA secretion, while suppressing hepatic BA synthesis through the downregulation of CYP7A1 (111). For ANIT-induced cholestasis, geniposide (25, 50 and 100 mg/kg) restores BA homeostasis by dual modulation of uptake and efflux: it represses basolateral OATP2-mediated uptake while inducing canalicular BSEP and basolateral OSTβ-dependent excretion, shifting BA elimination to urine. These actions occur alongside activation of hepatoprotective nuclear receptors (FXR, PXR, SHP), without altering detoxification enzymes (CYP3A2, UGT1A1, SULT2A1) (112, 113).

#### 4.2.2 Bile salt export pump (BSEP)

BSEP, a canalicular ATP-binding cassette (ABC) transporter, plays a crucial role in hepatobiliary bile acid secretion (114). By actively exporting bile salts from hepatocytes into bile canaliculi, BSEP sustains the enterohepatic circulation and prevents the accumulation of cytotoxic bile acids within the liver (115). Dysfunction of BSEP is a pivotal factor in the pathogenesis of cholestatic liver disease (116). Early research by Wu et al. (117) demonstrated that geniposide (150 mg/kg) significantly upregulates both mRNA and protein expression of the BSEP in cholestatic rats. This effect is mediated primarily through the activation of the FXR pathway. Specifically, geniposide promotes FXR binding to the BSEP promoter and facilitates the recruitment of the transcriptional co-activators PGC-1α and CARM1. This coordinated action markedly enhances BSEP transcription. In another investigation conducted by Chen et al. (118), geniposidic acid (25, 50 and 150 mg/kg) demonstrates dose-dependent hepatoprotective effects against ANIT-induced cholestasis and liver injury. Geniposidic acid pretreatment effectively restores bile flow, normalizes bile acid and bilirubin levels, reduces serum markers of liver damage (GOT, GPT, γ-GT, TB, DB, TBA), and mitigates histopathological damage. Importantly, geniposidic acid counteracts the ANIT-induced downregulation of FXR, BSEP, and Mrp2, significantly upregulating their mRNA expression.

#### 4.2.3 STAT3 and NF-κB signaling

NF-κB controls the transcription of genes linked to immune response, inflammation, cell fate, and activity (119). When NF-κB is activated, it leads to an increase in inflammatory factors like TNF-α, IL-1β, and IL-6, and TNF-α can subsequently activate NF-κB signaling (120). STAT3 is a cytoplasmic transcription factor that is part of the STAT family, which stands for signal transducers and activators of transcription. STAT3 activation can occur through various cytokines and growth factors, such as the typical IL-6. Consequently, NF-κB and STAT3 signaling are closely linked and play a significant role in hepatotoxicity (121). In an early study, Chen et al. (122) found that geniposide (50 mg/kg) significantly alleviated ANIT-induced cholestasis and liver injury by normalizing the expression of genes involved in bile acid metabolism and transport. Furthermore, geniposide reduced the levels of TNF-α and suppressor of cytokine signaling 3 (SOCS3), while also inhibiting the activation and expression of STAT3 and NF-κB.

### 4.3 Effects of iridoids on liver fibrosis (LF)

#### 4.3.1 Oxidative stress and flammatory

Oxidative stress and inflammation are critical factors driving the progression of hepatic fibrosis. Chronic inflammation exacerbates fibrosis severity by activating HSCs, which subsequently secrete pro-inflammatory cytokines such as TNF-α, IL-1β, and IL-6 to promote fibrogenesis (123). Concurrently, oxidative stress contributes to fibrotic pathogenesis through HSC activation and collagen deposition (123, 124). Emerging research highlights the potential of iridoids in mitigating these processes. Specifically, Lamiophlomis Herba demonstrates protective effects against LF, inflammation, and oxidative stress. These actions are partially mediated by its bioactive constituent shanzhiside methyl ester (SME), which significantly downregulates key fibrotic markers including fibronectin, collagen isoforms (Col1a1, Col3a1, Col-IV), α-SMA, laminin (LN), and procollagen type II (PC-II) (125). At the same time, research conducted by Ge et al. (126) demonstrated that SME upregulates antioxidant genes (*Nqo1, Ho1*) while simultaneously downregulating inflammatory genes (*Il-6, Il-18*), resulting in a subsequent decrease in the expression of genes and proteins related to the extracellular matrix, such as Col1a1, Col3a1, LN, α-Sma, PC-III, and Col-IV. In addition, geniposide (50 mg/kg) has been found to ameliorates CCl_4_-induced LF in mice by suppressing oxidative stress and inflammation. This is evidenced by enhanced activities of antioxidant enzymes (SOD, GSH-Px), reduced MDA levels, and reduced production of pro-inflammatory cytokines (IL-6, IL-1β, TNF-α) in liver tissue (127). Further study suggested that geniposide (25 and 50 mg/kg) restored BA profiles and suppress NLRP3 inflammasome activation, thereby alleviating LF in BDL mice, highlighting its potential for treating cholestatic liver fibrosis (128).

#### 4.3.2 Sonic hedgehog (Shh) signaling pathway

The Shh signaling pathway plays a vital role in embryonic development, tissue structuring, and the upkeep of stem cells. It regulates cell growth, specialization, and programmed cell death across different tissues (129). Notably, recent evidence suggests that Shh signaling plays a role in the activation of HSCs, indicating its potential as a novel therapeutic target for LF (130). In this regard, geniposide has demonstrated the ability to inhibit activated HSC-T6 cells, decreasing their viability with IC_50_ values of 77.11 and 42.88 μM at 24 and 48 h, respectively, and causing cell cycle arrest at the G2/M phase, and prominently suppressing the Shh signaling pathway (131).

#### 4.3.3 Transforming growth factor-β (TGF-β) signaling pathway

HSCs, which are nonparenchymal perisinusoidal cells located in the liver, have various functions and are crucial in the development of LF (77). TGF-β1 acts as a master regulator of fibrogenesis, promoting HSC activation. This activation occurs primarily through TGF-β1-induced phosphorylation of Smad2/3, leading to the formation and nuclear translocation of Smad complexes. Within the nucleus, these complexes upregulate key profibrotic genes, including those encoding α-SMA and COL1A1 (132). Significantly, geniposide exhibits strong antifibrotic properties by inhibiting LX-2 cell proliferation and reducing the expression of COL1A1, fibronectin, α-SMA, and TGF-β1/Smad signaling proteins (20 μM). These effects are confirmed *in vivo* (40 mg/kg), where geniposide significantly reduces CCl_4_-induced LF, HSC activation, and TGF-β1/Smad protein expression in mice (133).

### 4.4 Effects of iridoids on inflammation

#### 4.4.1 NOD-like receptor protein 3 (NLRP3) inflammasome

The NLRP3 inflammasome, the most extensively characterized inflammasome, is a cytoplasmic multiprotein complex composed of apoptosis-associated speck-like protein (ASC) and the effector pro-caspase-1 (134). The activation of NLRP3 is triggered by different stimuli, such as pathogens and non-infectious damage, primarily through the production of ROS from mitochondria or the activation and deubiquitination of cathepsin B (135). When stimulated, NLRP3 connects with ASC to form the inflammasome complex, resulting in the activation of caspase-1, which then proteolytically activates IL-1β and IL-18 (136). Notably, NLRP3 inflammasome activation is implicated in cholestatic liver injury, as observed in patients and murine models (137, 138). Critically, iridoids from GF targeting NLRP3 exhibit therapeutic potential against liver pathologies. Geniposidic acid (25, 50 and 100 mg/kg) acts as a covalent NLRP3 inhibitor that directly binds NLRP3, suppressing bile acid-induced inflammation in hepatocytes/macrophages and attenuating cholestatic injury in ANIT-induced models (139). In addition, genipin (25, 50 and 100 mg/kg) inhibits necroptosis-mediated NLRP3 activation by reducing RIP1/RIP3 necrosome formation, thereby suppressing caspase-1 cleavage and IL-1β/IL-18 release in GalN/LPS-induced hepatotoxicity (140). Furthermore, gardenoside combined with 6,7-Dimethoxycoumarin and Rhein directly attenuates hepatic NLRP3 inflammasome activation, ameliorating pathological features of NAFLD (141). These findings highlight the therapeutic potential of structurally related iridoids in modulating NLRP3-driven liver pathologies.

#### 4.4.2 Nuclear factor-κB (NF-κB) signaling pathway

The NF-κB signaling pathway is essential for controlling immune and inflammatory responses and is crucial in various physiological and pathological conditions, such as cancer, metabolic disorders, and neurodegenerative diseases (142, 143). NF-κB comprises a family of transcription factors that, upon activation, translocate to the nucleus to regulate the expression of genes involved in inflammation, immune response, cell proliferation, and survival (144). According to Liang et al. (145), gardenoside (10 and 20 μM) helps reduce cellular steatosis in HepG2 cells that have been induced with free fatty acids (FFA), representing a model of hepatic steatosis. This effect is associated with reduced supernatant levels of pro-inflammatory cytokines (TNF-α, IL-1β, IL-6) and the inhibition of NF-κB activation. Another investigation revealed that genipin (100 μM) can reduce inflammation in liver cells by inhibiting NF-κB activation, which in turn reduces the levels of important inflammatory mediators such as inducible nitric oxide synthase (iNOS) and TNF-α (146).

#### 4.4.3 Phosphoinositide 3-kinase/protein kinase B/mammalian target of rapamycin (PI3K/Akt/mTOR) signaling pathway

The PI3K/Akt/mTOR signaling pathway is a critical regulator of diverse cellular processes, including inflammation. Upon activation, Akt rapidly transmits signals to mTOR; the subsequent activation of mTOR exerts anti-inflammatory and anti-apoptotic effects (147, 148). Recent research has established a strong association between dysregulation of this pathway and the pathogenesis of liver diseases (149, 150). Supporting its therapeutic relevance, studies as early as 2017 demonstrated that geniposide (5, 10 and 20 mg/kg) protects rats against hepatic ischemia/reperfusion (I/R) injury. This protective effect is mediated, at least in part, through the suppression of inflammation and apoptosis via activation of the PI3K/Akt/mTOR signaling pathway, highlighting its potential as a modulator of this cascade (147).

#### 4.4.4 Methyl-CpG binding protein 2 (MeCP2) signaling pathway

MeCP2 orchestrates diverse aspects of gene expression regulation, encompassing transcriptional activation and repression, RNA splicing, chromatin remodeling, and modulation of chromatin architecture (151). Dysregulation of MeCP2 has been implicated in liver disease pathogenesis. Emerging evidence indicates that MeCP2 plays a central role in the activation of HSC_S_ (152). Notably, studies using mice model of CCl_4_-induced acute liver injury (20 mg/kg) and LPS-treated THP-1 cells (80 μM) suggest that geniposide may function as an effective modulator of the MeCP2-Hedgehog signaling axis during inflammatory pathogenesis (153).

#### 4.4.5 Mitogen-activated protein kinase (MAPK) signaling pathway

MAPK signaling pathway represents a critical intracellular cascade that regulates fundamental cellular processes—including proliferation, differentiation, and stress responses (154). This pathway comprises three principal subfamilies: extracellular signal-regulated kinases 1/2 (ERK1/2), c-Jun N-terminal kinases (JNK), and p38 MAPK. Upon extracellular stimulation, phosphorylation of ERK, JNK, and p38 activates these kinases to mediate inflammatory cascades (155). Within this mechanistic framework, geniposide (20 and 40 mg/kg) demonstrates hepatoprotective and regenerative effects in rat liver models, primarily through suppression of MAPK signaling. Crucially, geniposide specifically inhibits p38 MAPK phosphorylation (p-p38), thereby attenuating inflammatory pathway activation and subsequent hepatocellular apoptosis (156).

### 4.5 Effects of iridoids on oxidative stress

Oxidative stress, characterized by an imbalance between reactive oxygen species (ROS) production and endogenous antioxidant defenses, represents a central pathophysiological mechanism in the pathogenesis and progression of diverse liver diseases (157). Under pathological conditions, excessive hepatic ROS generation overwhelms both enzymatic and non-enzymatic antioxidant systems. Consequently, oxidative stress constitutes a key underlying driver in chronic liver disease (CLD) of various etiologies and is critically implicated in hepatocarcinogenesis (158). Currently, numerous studies have demonstrated that geniposide offers protective effects against various chemically induced liver injuries, such as those caused by acetaminophen (APAP, 10, 30 and 100 mg/kg) (159), tripterygium glycosides (TG, 20, 40 and 80 mg/kg) (159), and ethanol (20, 40 and 80 mg/kg) (160, 161), mainly by modulating oxidative stress pathways. Its mechanisms include: (a) restoration of redox homeostasis: elevating hepatic glutathione (GSH) levels and enhancing the activities of key antioxidant enzymes, including glutathione peroxidase (GPx), glutathione S-transferase (GST), superoxide dismutase (SOD), and catalase (CAT), across multiple injury models (159–161). (b) Suppression of pro-oxidant drivers: downregulating cytochrome P450 2E1 (CYP2E1) expression (thereby reducing APAP bioactivation) and attenuating lipid peroxidation [as evidenced by reduced malondialdehyde (MDA) and lipid peroxidation (LPO) products] (159–161). (c) Transcriptional regulation: upregulating the expression of antioxidant genes, such as CuZn-SOD and catalase (CAT), particularly in models of alcohol-induced liver injury (161). (d) Systemic metabolic rebalancing: ameliorating ethanol-induced disturbances in systemic metabolism, notably within amino acid metabolism pathways and oxidative stress biomarkers (162).

### 4.6 Effects of iridoids on autophagy

Autophagy is a critical catabolic process essential for maintaining hepatic homeostasis. It achieves this by degrading misfolded proteins, damaged organelles, and lipid droplets (163). This pathway is primarily regulated by autophagy-related genes (ATG), alongside key signaling pathways including mTOR, AMPK, and PI3K/AKT (164). Furthermore, the microtubule-associated protein 1A/1B light chain 3 (LC3) plays a pivotal role in autophagosome formation and governs the expression of lysosomal membrane proteins (LAMP-1, LAMP-2, RAB7), as well as facilitating autophagosome-lysosome fusion. Impairments in autophagic function are strongly associated with various forms of liver injury, while enhancing autophagy activation offers a potential strategy to mitigate disease progression (165). In this context, genipin (1, 2.5 and 5 mg/kg) effectively protects against sepsis-induced liver injury in CLP models by restoring impaired hepatic autophagic flux. It reduces liver damage, lowers serum aminotransferases and pro-inflammatory cytokines, and improves survival. Mechanistically, genipin upregulates the Atg12-Atg5 conjugate, restores Atg3 expression, normalizes LC3-II and p62 levels, and enhances lysosomal function by recovering LAMP-2 and Rab7 expression (166).

### 4.7 Effects of iridoids on mitochondrial

Mitochondria, which are crucial for ATP production and maintaining cellular balance, experience major dysfunction during reperfusion injury, leading to energy failure, excessive ROS production, calcium overload, and eventually cell death (167). Mitochondrial quality control (QC) maintains peak performance by coordinating biogenesis, fission and fusion dynamics, and mitophagy (168). Critically, the interplay between mitochondrial QC and oxidative stress constitutes a key pathogenic determinant in ischemic diseases (169). As early as 2017, the research of Shin et al. (170) indicated that genipin (100 mg/kg) ameliorated hepatic ischemia/reperfusion (IR) injury by restoring mitochondrial QC. Specifically, genipin reversed IR-induced mitochondrial dysfunction and oxidative stress through coordinated regulation of key QC pathways: it suppressed pathological fission (via Drp1, PINK1) while concurrently restoring compromised mitochondrial biogenesis (PGC-1α, NRF1, TFAM), mitophagy (Parkin), fusion (Mfn2), and energy-sensing pathways (SIRT1, p-AMPK).

### 4.8 Effects of iridoids on HCC

HCC is a predominant contributor to global cancer-related mortality, representing a substantial burden on healthcare systems (171). Surgical resection remains the primary treatment modality for patients with early to intermediate-stage HCC. Nonetheless, postoperative recurrence is observed in up to 70% of patients within 5 years, significantly undermining the long-term prognosis following hepatectomy (172, 173). Recent research has highlighted the therapeutic potential of iridoids derived from GF in the management of HCC. For instance, geniposide (30 mg/kg *in vivo* or 200 μg/ml *in vitro*) has been demonstrated to directly target the TLR4/MyD88 signaling pathway, thereby inhibiting VEGF expression and angiogenesis independently of HIF-1α (174). Additionally, genipin (25 and 50 mg/kg) exhibits anti-tumor activity through multiple mechanisms, including the activation of PPARγ, which impedes CCR2-mediated macrophage infiltration into the postoperative liver and reduces recurrence, as well as the inhibition of IRE1α in tumor-associated macrophages (175). Moreover, the combination of geniposide and paeoniflorin has been shown to enhance the anti-liver cancer effects of sorafenib by attenuating the activation of the NF-κB/HIF-2α/SerpinB3 pathway (176).

### 4.9 Others

Numerous studies confirm the therapeutic benefits of GF and its components, but high doses can cause liver toxicity (177–179). Geniposide, a major GF component, shows dose-dependent liver toxicity in rats, making it a suspected primary toxicant (180–183). However, new evidence points to genipin as the main toxicant. Luo et al. (179) found that pyrimidine, purine, amino acid metabolism, and pantothenate and CoA biosynthesis were disrupted in HepG2 cells treated with genipin, which suggests the potential hepatotoxicity of genipin. Furthermore, Wang et al. (184) found genipin's LD_50_ in mice to be 510 mg/kg, showing dose-dependent liver toxicity at 125, 250, and 500 mg/kg. Proteomic analysis suggested this toxicity involves impaired UDP-glucuronosyltransferase (UGT) and cytochrome P450 (CYP450) enzyme function. Supporting this, Huang et al. (185) implicated genipin's covalent binding to cellular proteins, particularly its inhibition of drug-metabolizing enzymes, in GF-induced hepatotoxicity. They confirmed that direct covalent binding and metabolic activation mediate genipin -induced CYP450 inactivation. Notably, Gao et al. (186) linked geniposide hepatotoxicity to its metabolite genipin, attributed to genipin's reactive hemiacetal structure. Exposure of the C-1 hydroxyl group facilitates covalent binding, specifically phase II conjugation with lysine residues, forming genipin-lysine (GP-LYS) adducts. These adducts contribute to cellular oxidative stress and subsequent hepatotoxic cascades.

## 5 Conclusions and future prospects

**Figure 2:**
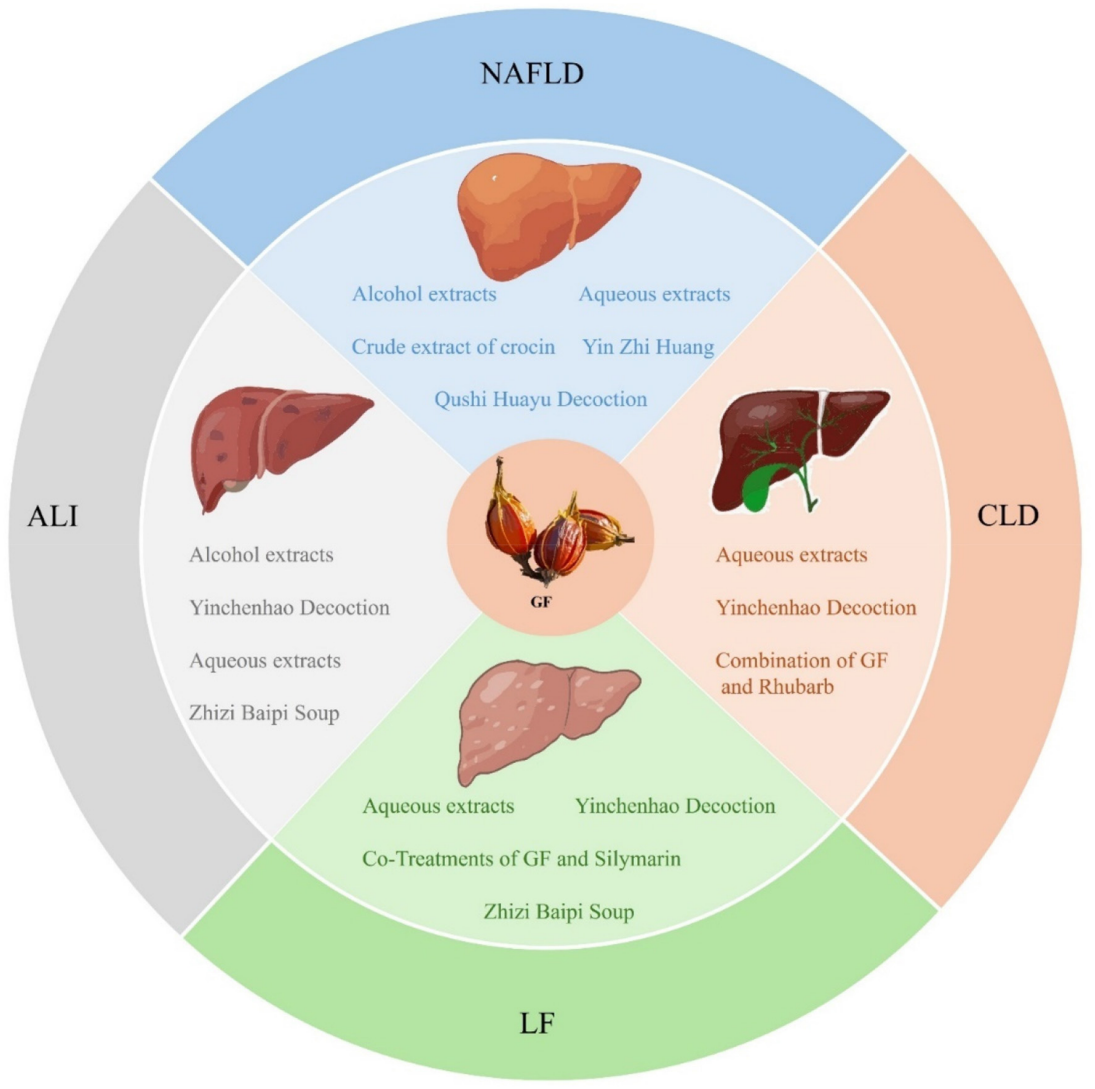
The therapeutic effects of GF extracts against NAFLD, CLD, ALI, and LF.

**Figure 3:**
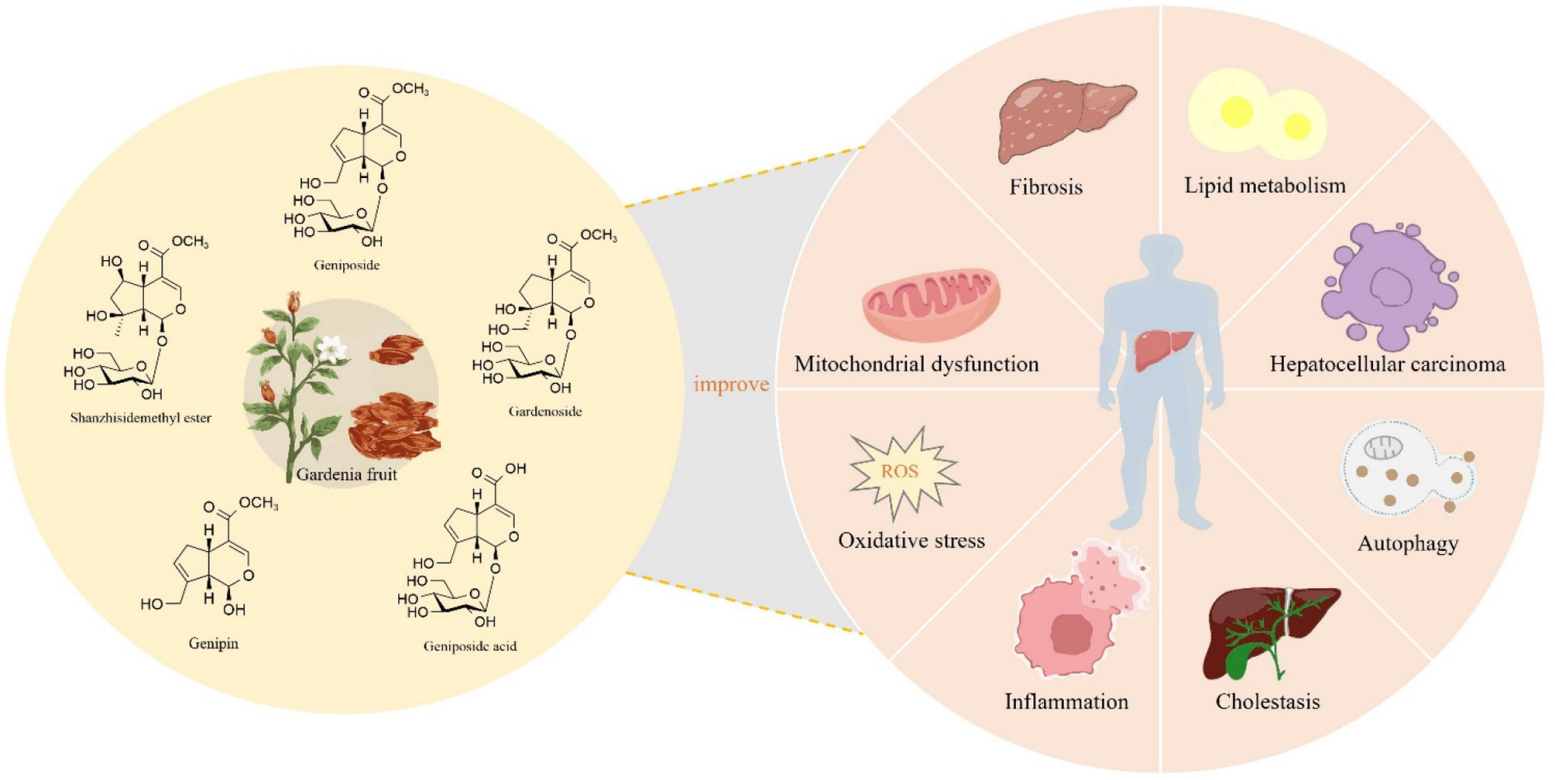
Iridoids in GF exert hepatoprotective effects, potentially through the modulation of lipid metabolism, cholestasis, inflammation, oxidative stress, mitochondrial function, autophagy, fibrosis, and hepatocarcinogenesis.

**Figure 4:**
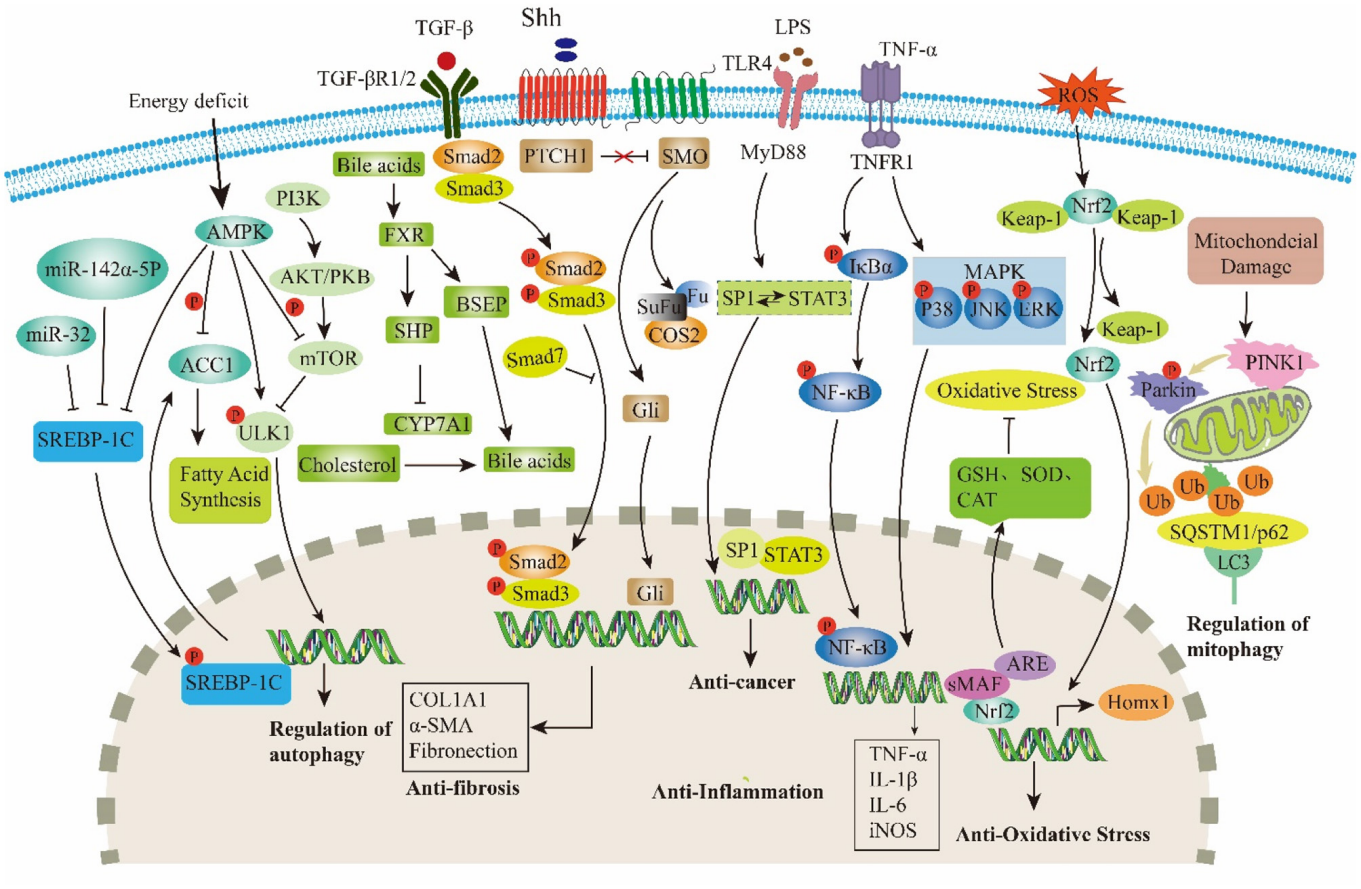
Major targets and signaling pathways modulated by iridoids in GF.

GF serves not only as a TCM but also as an innovative food resource with extensive application potential. With the ongoing advancements in modern medicine, a growing body of research has highlighted the indispensable role of GF in the treatment of liver diseases. Among its constituents, iridoids are identified as the primary active compounds responsible for its hepatoprotective properties, thereby reinforcing the medicinal value of GF. Therefore, this review systematically summarized 77 types of iridoids that have been isolated and identified from GF to establish the material basis of their hepatoprotective activity (Figure 1, Table 1). Then, preclinical animal experiments have confirmed that GF exerts significant therapeutic effects on various liver pathological models, such as NAFLD, CLD, ALI, and LF (Figure 2, Table 2). Finally, through data analysis, it was revealed that iridoids in GF exert hepatoprotective effects via multiple pathways, including regulation of lipid metabolism, cholestasis, fibrosis, inflammatory response, oxidative stress, autophagy balance, mitochondrial function, and hepatocarcinogenesis (Figures 3, 4, Table 3).

**Table 2:** GF is utilized in treating various liver diseases.

**Liver diseases**	**Models**	**Extract/medicines containing GF**	**Dosage**	**Effects**	**References**
NAFLD	HFD-induced SD rat model	Alcohol extracts	25, 50, 100 mg/kg, 6 weeks	TC↓, TG↓, FFA↓, ALT↓, AST↓, LDH↓, MDA↓, SREBP-1c↓, FAS↓, PPARα↑, PPARγ↓, AMPK↑, CPT-1↑	(59)
NAFLD	HFD-induced C57BL/six mice model	Aqueous extracts	28 mg/kg/100 μl/day, 8 weeks	mTOR↓, 8-OHdG↓, TGF-β↓, p-ERK↓	(60)
NAFLD	HFD-induced SD rat model	Crude extract of crocin	100, 200 mg/kg, 12 weeeks	TLR4↓, Myd88↓, NF-κB↓	(61)
NAFLD	HFD-induced C57BL/6J mice model	Yin Zhi Huang	10, 30 ml/kg, 16 weeks	TG↓, TC↓, ALT↓, AST↓, p-AMPK/AMPK↑, SREBP-1↓, FAS↓, ACC↑, CPT1A↑, PPAR-α↑	(62)
NAFLD	HFD-induced C57BL/six mice model	Qushi Huayu decoction	0.465, 0.93, 1.86 g of crude drug/ml, 10 ml/kg body weight, daily, 2 weeks	XBP1s↓, p-IRE1α↓, ACC1↓, ACC2↓, FAS↓, SCD1↓, GPAT1↓, AGPAT↓, PAP↓, DGAT2↓	(63)
CLD	ANIT-induced SD rat model	Aqueous extracts	21, 42 mg/kg, 5 days	Cyp8b1↑, Fxr↑, Ntcp↑, Oatp1↑, Hepatic TaurineBA↓, Fecal PBA Excretion↑	(67)
CLD	ANIT -induced SD rat model	Aqueous extracts	4 g/kg, 7 days	Hepatic TaurineBA↓, Fecal PBA Excretion↑, MDA↓, TBA↓ TBIL↓, SOD↑	(68)
CLD	ANIT-induced SD rat model	Yinchenhao decoction	6, 9, 12 g/kg	TBA↓, UGT1A1↓, MRP2↓, BSEP↓, OCT1↓, NTCP↓, MDR1, OATP1A2/4↑, MATE1↓	(69)
CLD	ANIT-induced SD rat model	Combination of GF and Rhubarb	2 g/kg 7 days	TBIL↓, DBIL↓, ALT↓, AST↓	(70)
ALI	APAP-induced ICR mice model	Alcohol extracts	0.44, 0.88 g/kg	ALT↓, AST↓, TNF-α↓, IL-6↓, IL-1β↓, GSH↓ MDA↓, CYP2E1↓	(72)
ALI	CCl_4_-induced SD rat model	Yinchenhao decoction	8 g/kg, 10 days	Firmicutes↓, bacteroidetes↓, ALT/AST↓	(73)
ALI	BCG and LPS-induced ICR mice.	Aqueous extracts	112.5, 225, 450 mg/ml, 10 days	ALT↓,AST↓, IL-2 ↑, IL-10↓, IL-4↓, TNF-γ↓	(74)
ALI	Con A-induced ICR mice model	Zhizi Baipi soup	20 mg/kg, 7 days	ALT↓, AST↓, SOD↑, MDA↓, IFN-γ↓, TNF-α↓, NF-κB-p65↓, NF-κB-p-]p65↓, IL-4↑, IL-6↑	(75)
LF	TAA-induced C57BL/six mice model	Aqueous extracts	200 mg/kg, 8 weeks	IL-1β↓, TNF-α↓, NF-κB p38↓, α-SMA↓, SIRT1↑, p-AMPKα↑, p-LKB1↑, NOX2 ↓, Nrf2/HO-1↑	(78)
LF	CCl_4_-induced SD rat model	Aqueous extracts	5, 10 and 20 g/kg, 4 weeks	AST/ALT↓, SOD↑, MDA↓, GSH↑, GSH-Px↑, α-SMA↓, ColIV↓	(79)
LF	DMN-induced SD rat model	Yinchenhao decoction	3.15 g/kg, 4 weeks	ALT↓, AST↓, ALP↓, TBA↓, TBIL↓Hepatic TaurineBA↓, Fecal PBA Excretion↑, BSEP↓, α-SMA↓, TGF-β1↓, p-Smad3↓, p-ERK1/2↓	(80)
LF	TAA-induced C57BL/six mice model	Co-treatments of GF and Silymarin	50, 100 mg/kg, 8 weeks	4-HNE↓, 8-OHdG↓, ROS↓, MDA↓, GSH↑, SOD↑, TNF-α↓, IL-1β↓, IL-6↓, α-SMA↓, ColT1↓, ColT3↓, TGF-β1R↓, Smad2↓, Smad3↓, Nrf2↑, HO-1↑, Sirtuin1↑	(81)
LF	CCl_4_-induced ICR mice model	Zhizi Baipi soup	2 weeks	Collagen I↓, α-SMA↓, TGF-β↓	(82)

**Table 3:** The role and mechanism of iridoids from GF in the treatment of liver diseases.

**Experimental model**	**Iridoids**	**Dosage**	**Molecular mechanisms**	**References**
Nrf2^−/−^C57BL/six mice; OA and PA-induced HepG2 cells	Geniposide	50, 75 and 100 mg/kg	Inhibit Nrf2/AMPK/mTOR signaling pathway	(86)
HFD-induced ICR mice model	Mixture of peanut skin extract, geniposide, and isoquercitrin	/	Regulate the TLR4/NF-κB, AMPK/ACC/CPT1, and AMPK/UKL1/LC3B signaling pathways	(90)
HFD-induced obese mice model	Genipin	5 and 20 mg/kg	Regulation miR-142a-5p/SREBP-1c axis	(93)
HFD-induced obese mice model	Genipin	20 mg/kg	Inhibit miR-132 expression	(94)
HFD-induced C57BL/six mice model	Combination of geniposide and chlorogenic acid	/	Reduce the signaling of gut-derived lipopolysaccharide (LPS); inhibit RhoA/ROCK signaling	(98)
HFD-induced C57BL/six mice model; FXR^−/−^ mice	Combination of geniposide and chlorogenic acid	/	Improve the gutmicrobiome; activate FXR signaling	(99)
PA and LPS-induced AML12 cells model; HFD-induced C57BL/6J mice model	Gardenoside	10, 25 and 50 μM *in vitro* and 40 mg/kg *in vivo*	Negative-regulate pyroptosis markers (NLRP3, ASC, caspase-1 p20, GSDMD-N, IL-1β); reduce CTCF/DPP4 expression	(103)
HFD-induced obese mice model; Hepatocytes treated by free fatty acids	Genipin	/	Inhibit UCP2-mediated pyroptosis.	(104)
Hepatocytes treated by H^2^O^2^	Geniposide	900 μg/ml	Regulate the global DNA methylation level; inhibit the expression of P53, Bcl-2 and Akt	(106)
HFD-induced C57BL/6 and ApoE^−/−^ mice model; HepG2 cells and Caco2 cells	Geniposide	50 mg/kg	Regulate the liver-gut crosstalk of bile acids mediated by FXR; promote hepatic bile acid synthesis and ileal bile acid excretion via regulating the enterohepatic	(110)
DDC-induced sclerosing cholangitis mice model	Geniposide	50 and 100 mg/kg	Inhibit expressions of CK19 and Ki67; up-regulate canalicular efflux transporters (BSEP, MRP2, MDR1, MDR2); reduce CYP7A1 mRNA expression	(111)
ANIT-induced IC rat model	Geniposide	25, 50 and 100 mg/kg	Modulate the bile secretion pathway and the glutathione pathway; regulate the expression of ABCG5, NCEH1, OAT3, and GST	(112)
ANIT-induced IC rat model	Geniposide	/	Reduce basolateral bile acids uptake via repression of OATP2; decrease Bile acids biosynthesis through down-regulation of CYP7A1, CYP8B1, and CYP27A1; enhance mRNA level of basolateral transporter OSTβ; activate FXR, PXR, and SHP.	(113)
ANIT-induced ICR mice model; The HepG2 cell line	Geniposide	150 mg/kg	Promote the expression of BSEP through activate FXR and Nrf2 signaling pathway.	(117)
ANIT-induced liver injury with acute intrahepatic cholestasis SD rat model	Geniposidic acid	25, 50 and 150 mg/kg	Up-regulate mRNA expression of FXR, BSEP and Mrp2	(118)
ANIT-induced liver injury ICR mice model	Geniposide	50 mg/kg	Inhibit STAT3 and NF-κB signaling pathway	(122)
SD rats and HSC-T6 cells	shanzhiside methyl ester	/	Inhibit Nrf2/NF-κB signaling pathway	(126)
CCl_4_-induced C57BL/six mice model	Geniposide	50 mg/kg	Reduce oxidative stress and inflammatory respose, inhibit apoptosis and modulate overall metabolism	(127)
C57BL/six mice model	Geniposide	25 and 50 mg/kg	Active SIRT1/FXR signaling	(128)
CCL_4_-induced C57BL/six mice model HSC-T6 cells	Geniposide	/	Inhibit Shh signaling, inhibits the activation of HSC	(131)
TGF-β1-induced LX-2 cell CCl_4_-induced BALB/c mice model	Geniposide	40 mg/kg *in vivo*	Inhibit TGF-β1/Smad signaling	(133)
ANIT-induced C57BL/six mice NLRP3^−/−^ mice	Geniposidic acid	25, 50 and 100 mg/kg	Inhibit NLRP3 inflammasome activation	(139)
GalN/LPS-induced ICR mice model	Genipin	25, 50 and 100 mg/kg	Inhibit necroptosis-mediated inflammasome signaling	(140)
HFD-induced rats model	Gardenoside combined with 6,7-Dimethoxycoumarin and Rhein	/	Inhibit the NLRP3 inflammasome	(141)
FFA- induced HepG2 hepatocytes	Gardenoside	10 and 20 μM	Inhibit NF-κB activity	(145)
IL-1β-stimulated hepatocytes	Genipin	100 μM	Inhibit NF-κB activity	(146)
Hepatic IRI injury (abdominal surgery without occlusion to produce an ischemia)	Geniposide	/	Inhibit PI3K/Akt/mTOR signaling pathway.	(147)
CCl_4_-induced C57BL/six mice model LPS-treated THP-1 cells	Geniposide	/	Inhibit MeCP2-Hedgehog signaling axis	(153)
CCl_4_-induced rats models	Geniposide	20 and 40 mg/kg	Inhibit p38 MAPK signaling pathway	(156)
APAP-induced C57BL/six mice model	Geniposide	20, 40 and 80 mg/kg	Inhibit CYP 2E1 and attenuate the exhaustion of GSH and accumulation of MDA Inhibit TLR 4/NF-κB signaling pathway	(159)
Tripterygium glycosides (TG)-induced Kunming mice model	Geniposide	20, 40 and 80 mg/kg	Promote GSH, GST, GPX, SOD, CAT	(160)
Alcohol-induced Kunming mice model	Geniposide	20, 40 and 80 mg/kg	Promote GSH, GST, GPX, CuZn-SOD and CAT Levels	(161)
Alcohol-induced konmin mice model	Geniposide	/	Improve abnormal metabolism, alleviate disorders related to amino acid metabolism and oxidative stress	(162)
sepsis-induced mice	Genipin	1, 2.5 and 5 mg/kg	Restore impaired autophagic flux	(166)
C57BL/six mice (ischemia/reperfusion)	Genipin	100 mg/kg	Modulating mitochondrial quality control	(170)
Orthotopic mouse model Human HCC cell line PLC/PRF/5 cells and HUVECs	Geniposide	30 mg/kg *in vivo* or 200 μg/ml *in vitro*	Inhibit TLR4/MyD88 signaling pathway	(174)
The C57BL/six mice, BALB/c nude mice and NOD-SCID mice Luciferase-tagged MHCC-97L cells	Genipin	25 and 50 mg/kg	Activate PPARγ	(175)
H22 hepatoma tumor-bearing mouse model H22 cell line	Mixture of geniposide, paeoniflorin combined with sensitized sorafenib	/	Activate NF-κB/HIF-2α/SerpinB3 signaling pathway	(176)
SD rats HepG2 cells	Genipin	/	Distrubed pyrimidine, purine, amino acid metabolism, and CoA biosynthesis	(179)
ANIT-induced Kunming mice model	Genipin	125, 250, and 500 mg/kg	Impaire UDP-glucuronosyltransferase system and cytochrome P450 enzyme activity.	(184)
Male SD rats	Genipin	/	Induce P450 inactivation	(185)
C57BL/six mice HepG2 cells	Geniposide	/	attributed to genipin's reactive hemiacetal structure	(186)

Despite the progress in existing research, several challenges and difficulties persist: (1) the majority of studies on GF extracts are predominantly based on *in vitro* cell experiments and rodent models, with a notable lack of human clinical evidence. To elucidate the efficacy and safety of GF in promoting human health, there is an urgent need for high-quality clinical trials that can provide robust scientific support. (2) Although GF exhibits diverse biological activities and holds significant medicinal value, making it a promising candidate for health diets and drug development, its current applications in health-related fields remain relatively limited. The development of functional foods and health products derived from GF is still in its nascent stages, necessitating further exploration of its potential value. (3) The number and types of iridoids isolated from GF are limited, particularly with respect to low-abundance analogs. To facilitate the efficient enrichment and large-scale preparation of these compounds, advanced technologies such as synthetic biology and biocatalysis should be employed to enhance structural diversity and functional research. (4) Current research on GF iridoids has predominantly focused on major constituents such as geniposide, genipin, gardenoside, geniposidic acid, and shanzhiside methyl ester. These compounds are not only present in relatively high abundances but also more readily isolated and quantified, thereby facilitating comprehensive pharmacological evaluation both *in vitro* and *in vivo*. In contrast, studies on minor or low-abundance iridoids have largely been restricted to phytochemical identification and structural elucidation, with limited subsequent investigation into their biological activities. We propose that the scarcity of conclusive pharmacological data on these low-abundance compounds may stem from the practical challenges associated with obtaining sufficient quantities of purified material—particularly for *in vivo* studies. Moreover, it is plausible that these minor iridoids exhibit distinct pharmacokinetic profiles or higher bioactivity, potentially contributing to therapeutic effects through unique mechanisms. However, validation of these hypotheses remains challenging due to the limited availability of purified compounds and the current inadequacy of sensitive analytical methodologies for tracing their metabolic fate and tissue distribution. Further research is essential to elucidate the contributions of these understudied iridoids to the hepatoprotective effects of GF and to clarify their underlying molecular mechanisms. (5) Despite the extensive application of GF in clinical practice, its safety profile remains incompletely characterized, raising concerns about potential risks associated with its use. Recent studies have indicated that high doses of GF may induce hepatotoxicity and nephrotoxicity. For instance, Li et al. (177) demonstrated that rats administered GF for 12 weeks exhibited impaired liver and kidney function following long-term or high-dose exposure. Furthermore, Luo et al. (179) reported that GF treatment resulted in significant plasma biochemical alterations and histopathological changes in the liver. Additionally, it disrupted multiple metabolic pathways, including purine and amino acid metabolism in the liver, and pyrimidine, primary bile acid, amino acid, pantothenate, and CoA biosynthesis pathways in the plasma. Further evidence suggests that geniposide and its aglycone metabolite genipin are the principal compounds responsible for the observed hepatotoxicity of GF (178). According to TCM principles, the synergistic combination of herbs can enhance therapeutic efficacy while reducing toxicity (187). Consequently, relying solely on the toxicity assessment of individual components, such as GF extract, to evaluate the overall safety of the herb is scientifically unsound and may lead to inaccuracies and concealed safety risks. In recent years, there has been a gradual increase in reports of hepatotoxicity linked to geniposide and genipin, underscoring the necessity of exploring emerging technologies, such as nano-sustained release systems, to mitigate their toxic effects and enhance clinical safety. (6) This review primarily examines the hepatoprotective properties of GF and its iridoid constituents. Notably, gardenia flowers and leaves also possess a variety of chemical compounds akin to those present in the fruit, suggesting the potential presence of novel active iridoids (188, 189). Consequently, these plant parts represent promising candidates for the development of new hepatoprotective drugs and merit further exploration. In summary, addressing the identified challenges could significantly expand the applicability of GF iridoids within both the pharmaceutical and food sectors.

## GLOSSARY

TC, total cholesterol; TG, triglyceride; FFA, free fatty acid; ALT, alanine aminotransferase; AST, aspartate aminotransferase; LDH, lactate dehydrogenase; MDA, malonaldehyde; SREBP-1C, sterol regulatory element binding protein-1c; FAS, Fas cell surface death receptor; PPARα, peroxisome proliferator-activated receptor α; PPARγ, peroxisome proliferator-activated receptor gamma; AMPK, adenosine5′-monophosphate (AMP)-activated protein kinase; p-AMPK, phospho5′AMP-activated protein kinase; CPT-1, carnitine palmitoyltransferase-1; mTOR, mammalian target of rapamycin; 8-OhdG, 8-hydroxy2′-deoxyguanosine; TGF-β, transforming growth factor-β; p-ERK, phosphorylated extracellular signal-regulated kinase; TLR4, toll-like receptor-4; Myd88, myeloid differentiation primary response gene 88; NF-κB, nuclear factor kappa-B; CPT1A, carnitine palmitoyltransferase 1A; p-IRE1α, phosphorylated inositol-requiring enzyme 1α; GPAT1, glycerol-3-phosphate acyltransferase 1; AGPAT, 1-acylglycerol-3-phosphate O-acyltransferase; PAP, prostatic acid phosphatase; DGAT2, diacylglycerol-O-acyltransferase homolog 2 Cyp8b1; Fxr, farnesoid X receptor; Ntcp, sodium taurocholate cotransporting polypeptide; Oatp1, organic anion transporting polypeptide 1; TBA, total bile acid; TBIL, total bilirubin; SOD, superoxide dismutase; HO-1, heme oxygenase 1; Nrf2, nuclear factor erythroid 2-related factor 2; Smad3, mothers against decapentaplegic homolog 3; p-Smad3, phospho- mothers against decapentaplegic homolog 3; Smad2, mothers against DPP homolog 2; ColT3, tumor (T3); α-SMA, α-smooth muscle actin; IL-2, interleukin-2; IL-6, interleukin-6; IL-4, interleukin-4; IL-10, interleukin-10; IL-1β, interleukin-1 beta; TNF-α, tumor necrosis factor-alpha; GSH, glutathione; ROS, reactive oxygen species; 4-HNE, 4-hydroxynonenal; p-ERK1/2, phosphorylated extracellular regulated kinase 1/2; BSEP, bile salt export pump; ALP, alkaline phosphatase; ColIV, collage typeIV; NOX2, NADPH oxidase 2; SIRT1, silent mating type information regulation 2 homolog-1; p38, P38 mitogen-activated protein kinase; IFN-γ, interferon-gamma; TNF-γ, tumor necrosis factor-γ; CYP2E1, cytochrome P450 2E1; DBIL, directbilirubin; MATE1, multidrug and toxin extrusion protein 1; MDR1, multidrug resistance 1; OATP1A2, organic anion transporting polypeptide 1A2; MRP2, multidrug resistance-associated protein 2; UGT1A1, uridine diphosphate-glucuronosyltransferase 1A1; FDA, food and drug administration; CCl_4_, carbon tetrachloride.
